# La place de l'imagerie par résonance magnétique dans le carcinome lobulaire du sein

**DOI:** 10.11604/pamj.2014.18.21.4055

**Published:** 2014-05-06

**Authors:** Wail Bouzoubaa, Meryem Laadioui, Fatime Zahra Fdili Alaoui, Sofia Jayi, Hakima Bouguern, Hikmat Chaara, Moulay Abdelilah Melhouf

**Affiliations:** 1 Service de Gynécologie Obstétrique II, CHU Hassan II, Fès, Maroc

**Keywords:** IRM, carcinome lobulaire, multifocalité, multicentricité, bilatéralité, MRI, lobular carcinoma, multifocal, multicentricity, bilateral

## Abstract

Le carcinome lobulaire reste une entité histologique peu fréquente du cancer du sein, toute fois la place qu'occupe le cancer du sein actuellement dans la cancérologie féminine, justifie la connaissance des particularités de ce type de cancer mammaire. Le diagnostic paraclinique est basée sur le couple écho-mammographie a la recherche de multifocalité, multicentricité ou bilatéralité, d'où l'intérêt de l'IRM qui est la technique la plus sensible pour la mise en évidence de ces lésions et qui est devenue un examen de pratique courante dans le carcinome lobulaire du sein. Par le présent travail, et sous la lumière de la revue de la littérature, nous allons essayer de dégager les aspects épidémiologiques, cliniques, et paracliniques, du carcinome lobulaire du sein, et insister sur les indications et l'intérêt de l'IRM dans la prise en charge de ce type histologique.

## Séminaire

Le cancer du sein est actuellement le cancer le plus fréquent chez la femme, et pose un véritable problème diagnostique et thérapeutique. 80 à 85% des cancers du sein sont représentés par des carcinomes canalaires. Parmi les 15 à 20% restants, la forme histologique la plus fréquente est représentée par les carcinomes lobulaires [1, 2].

On distingue le carcinome lobulaire infiltrant (CLI) qui constitue le deuxième type histologique du cancer mammaire avec une fréquence de 2,8 à 6%, et la forme lobulaire in situ (CLIS) qui ne représente que 0,7% [1–4], ce dernier est considéré par la plupart des auteurs comme un facteur de risque de cancer invasif plutôt qu´un état cancéreux.

Par rapport aux carcinomes canalaires in situ ou infiltrants, les carcino¬mes lobulaires in situ ou infiltrants s'individualisent par des particularités diagnostiques, thérapeutiques et évolutives, qui doivent être bien connues du gynécologue mais aussi du médecin généraliste qui pourrait être amené à participer au diagnostic et à la surveillance de ces patientes [5, 6].

L'imagerie par résonnance magnétique s'est imposée comme l'examen indispensable dans le diagnostique et dans le bilan d'extension locale du CLI, car elle permet d'une part, d'approcher de façon plus précise la taille réelle de la lésion, et d'autre part, de rechercher une multifocalité et une bilatéralité.

Le carcinome lobulaire représente 2,8% à 6% de l´ensemble des lésions malignes du sein. Il constitue, par sa fréquence, le deuxième type histologique après le cancer galactophorique (canalaire) dont la fréquence est de 85% [1, 2]. Le carcinome lobulaire in situ (CLIS) est défini (selon l'OMS) comme un carcinome intéressant les canalicules intra-lobulaires sans envahissement du tissu conjonctif voisin. Il est considéré comme un facteur de risque de cancer invasif plus qu´un état cancéreux. Sa fréquence varie selon les auteurs entre 0,8 et 3,8% [7, 8], et il est retrouvé sur 0,8 à 2% des biopsies réalisées pour lésions bénignes [8].

Les carcinomes lobulaires infiltrants représentent 4 à 10% des cancers du sein [5]. Leur originalité tient essentiellement à leur difficulté diagnostique et à leur particularité métastatique évolutive [3, 4, 6].

Les femmes avec CLIS sont généralement plus jeûnes que celles avec la forme infiltrante. L´âge moyen de survenu du CLIS est la quarantaine [9–11] et presque les deux tiers sont en phase préménopausique contrairement aux patientes avec un cancer infiltrant.

Plusieurs facteurs de risque sont incriminés dans l´apparition d´un cancer du sein, mais les données de la littérature ont confirmé que la prescription de traitements hormono-substitutifs (THS) est associée à un excès de risque de développer un carcinome lobulaires [12, 13], ainsi, dans la Million Women Study, le risque relatif de CLIS chez les utilisatrices par rapport aux femmes n'ayant jamais pris de THS étais de 2,82 alors qu'il n'est que de 1,56 pour le carcinome canalaire in situ [12–14].

En cytogénétique, une étude récente a montré que les lésions de CLIS ont de nombreuses expressions chromosomiques en comparaison avec celle rapportées pour les autres types de cancer du sein. Cette analyse a montré que la perte du 16q et le gain du 1p était l´altération chromosomique la plus détectée, dans les 17 cas de l’étude, 88% étaient positives, la plupart des cas présentaient un résultat anormal lors du test de configuration des E-cadhérine, ce qui confirme que la modification de la E-cadhérine contribue à la néoplasie phénotype dans les CLIS, suggérant qu´un mécanisme unique est responsable de ces deux changement dans certains cas au moins [15]. Une autre étude récente a montré que la perte du 16q et le gain du 1p était un facteur de risque d’évolution vers le carcinome invasif [11, 12]. Par ailleurs et par rapport aux carcinomes canalaires invasifs, les carcinomes lobulaires invasifs présentent des taux inférieurs de la surexpression de l´erb-B2 [15].

Sur le plan clinique le cancer lobulaire siège plus fréquemment dans le sein gauche que dans le sein droit, préférentiellement le quadrant supéro-externe (QSE) [16]. Dans une série publiée de 2017 patientes, le sein gauche est affecté chez 50% des patientes, le sein droit chez 39% et les deux seins chez 10,4% [16]. Lambioid et Shelly sur neuf mastectomies ont trouvés que la localisation la plus fréquente est au niveau du QSE (25%).

La multifocalité (foyers disséminés dans le même quadrant) est estimée en moyenne à 30% [10], elle est variable selon les auteurs [8–15]. Pour la multicentricité (foyers disséminés dans d´autres quadrants), elle est estimée en moyenne à 50% [10], elle est variable de 36% à 70% selon les auteurs [8–15]. Selinko [17] dans une série de 62 cas de CLI, a retrouvé la multifocalité et la multicentricité dans 21% des cas.

Le CLIS est bilatéral dans 30 à 35% des cas, alors que le carcinome lobulaire invasif est bilatéral dans 25% à 36% des cas, ce qui conduit certains auteurs à pratiquer de façon systématique une biopsie controlatérale en miroir [8–18]. Actuellement Le diagnostic de cancer du sein bilatéral peut être suspecté en dehors de toute expression clinique, lors de la découverte fortuite par écho-mammographie du sein controlatéral, dans le cadre du suivi des cas des cancers du sein unilatéraux traités ou dans le cadre d'une mammographie de dépistage, mais aussi il peut être suspecté devant des éléments cliniques évocateurs, notamment la palpation d'un nodule mammaire, la présence d'un écoulement mamelonnaire, d'une rétraction du mamelon ou la découverte d'une adénopathie axillaire. Le tableau diagnostique du cancer du sein bilatéral, le plus souvent rencontré dans la littérature, associe une lésion clinique d'un côté à une anomalie radiologique controlatérale dans 43,5% des cas [19, 20]. Cette dernière situation était également fréquente dans les deux séries détaillant les modalités diagnostiques avec des taux rapportés de 34% pour Polednak et al. [21] et 45% pour De La Rochefordière et al. [20]. Dans la série de Polednak et al. en 2003, 104 patientes parmi 300, soit 34,7% avaient un cancer du sein bilatéral et synchrone (CSBS) sans traduction clinique diagnostiqué à partir d'anomalies bilatérales à la mammographie. Dans celle de De La Rochefordière et al. en 1994, le taux de CSBS sans traduction clinique était seulement de 7%. Ces différences pourraient s'expliquer par la généralisation du dépistage par mammographie permettant un diagnostic plus précoce de lésions non palpables. En outre, la capacité diagnostique de la mammographie, accrue par les techniques de numérisation, pourrait également expliquer le faible taux de cancer du sein bilatéraux métachrones pour certains auteurs, cancers qui seraient diagnostiqués plus précocement [21].

Par sa grande sensibilité, supérieure à celle de la mammographie et de l’échographie, l'imagerie par résonnance magnétique s'est imposée comme l'examen indispensable dans le diagnostique et dans le bilan d'extension locale du CLI. Qayum a détecté jusqu’à 43% de faux négatifs mammographiques à IRM positives. L'intérêt de cet examen est, d'une part, d'approcher de façon plus précise la taille réelle de la lésion, et d'autre part, de rechercher une multifocalité et une bilatéralité. Dans les deux cas, la thérapeutique peut être modifiée. Cet intérêt est majeur dans le carcinome lobulaire infiltrant puisqu'il est plus souvent responsable de localisations multiples. On retrouve le plus souvent un syndrome tumoral malin sans caractère spécifique : masse régulière, prise de contraste rapide, intense avec plateau et lavage tardif du produit de contraste, il peut également s'agir de rehaussement moins marqué avec des cinétiques de prise de contraste intermédiaires [22, 23].

L'IRM a une excellente sensibilité dans le diagnostic de cancer du sein, supérieure à 90% pour les cancers invasifs [24–26]. En revanche, la spécificité est moyenne, avec des chiffres variant entre 40 et 80% ; cette variabilité est liée aux facteurs techniques de réalisation des IRM, aux critères d'interprétation utilisés, et surtout à la sélection des patientes incluses dans les études. Du fait de cette spécificité moyenne, pour garder une valeur prédictive positive acceptable, l'utilisation de l'IRM en dépistage ne peut être envisagée que dans la population de patientes ayant une prévalence de cancer élevée, et pour laquelle la mammographie est en difficulté [25–27].

L'IRM est la technique la plus sensible pour la mise en évidence de lésions multifocales ou multicentriques ou contro-latérales. Les limites de l'IRM sont : une prise de contraste retardée dans le temps à cause de la composante fibreuse des lésions in situ [22, 23].

Les aspects morphologiques des carcinomes lobulaires a l'IRM, sont décrits sous forme d´une masse ronde unique, d´une distorsion architecturale, d´un rehaussement micronodulaire segmentaire ou diffus et sous la forme de l´association d'une masse focale et d'un rehaussement régional, ainsi la majorité des carcinomes lobulaires infiltrants se réhaussent de façon hétérogène plutôt qu'homogène [22, 23].

Une étude rétrospective de N Fabre Demard en 2005 [24] portante sur 35 patientes âgées de 38 à 76 ans ayant toutes un carcinome lobulaire infiltrant, chaque dossier comportait une évaluation clinique au moment du diagnostic, une mammographie, une échographie mammaire et une IRM mammaire. Les 35 cancers étudiés présentaient tous un rehaussement à l'IRM. Ce rehaussement était nodulaire ou focal pour 24 patientes, en plage pour 10 patientes et diffus pour une patiente. La cinétique de rehaussement tumoral était suspecte de malignité (précoce, rapide, intense) chez 33 patientes. Pour 11 patientes le bilan d'extension était positif à l'IRM retrouvant au final 8 cancers. L'IRM avait induit 3 cas de biopsies pour des lésions bénignes mais a permis d'optimiser le geste chirurgical pour les 8 cas de cancers en élargissant le geste conservateur dans 3 cas, en transformant une indication de chirurgie conservatrice en mastectomie dans 3 cas et en permettant une biopsie-exérèse d'une lésion néoplasique controlatérale dans 2 cas.

Le diagnostic de cancer lobulaire, qui reste très difficile à établir avec la mammographie classique, est donc grandement simplifié par l'IRM, qui permet de mieux visualiser la multifocalité. Nous ne ferons donc pas l’économie de cet examen devant des aspects cliniques et mammographiques évocateurs de cancers lobulaires [23, 28].

De plus, une revue systématique publiée en 2007 [29, 30] a évalué les performances de l'IRM et de la mammographie pour dépister un cancer du sein chez des femmes à haut risque. Deux des 11 études analysées n'avaient inclus que des femmes porteuses d'une mutation BRCA1/2 [31, 32]. La probabilité de laisser une anomalie ACR4 ou plus après un examen négatif avec une prévalence de 2% est de 1,4% pour la mammographie seule et de 0,3% avec les 2 examens [32].

Ces dernières années il y a eu un élargissement des indications de l´IRM mammaire [29], et ceux-ci devant un cancer qui n'est pas détecté en imagerie standard, devant une taille tumorale qui est difficilement évaluable, devant une atteinte multifocale ou une atteinte pariétale suspectée, ou s´il existe une contre-indication à un traitement local complet, et enfin lorsqu´un traitement néoadjuvant est indiqué.

Une IRM mammaire systématique dans le cadre du bilan préoperatoire d´un cancer du sein n´est pas recommandée ce jour selon les recommandations du GNGOF 2011. Les indications de l´IRM mammaire avec des avantages potentiels sont (recommandation de la HAS et du CNGOF 2011) essentiellement devant des nouvelles patientes avec un diagnostic de cancer lobulaire infiltrant, patientes à haut risque de cancer du sein, patiente avec une discordance entre la clinique, la mammographie et l´échographie, patiente de moins de 40 ans et il faut savoir que l´IRM n´a pas de place devant un sein inflammatoire ou métastatique sauf pour évaluer le sein controlatéral.

Le carcinome lobulaire du sein reste peu fréquent par rapport au carcinome canalaire, toute fois il présente plusieurs particularités cliniques et radiologiques. Le diagnostic paraclinique est basée sur le couple écho-mammographie a la recherche de multifocalité, multicentricité ou bilatéralité, d'où l'intérêt de l'IRM qui est devenue un examen de pratique courante en pathologie mammaire, avec un élargissement de ces indications, même si le nombre d'appareils reste insuffisant pour la demande dans notre pays, sa place va en croissant pour améliorer la prise en charge de nos patiente.
